# Preserved walking function without postoperative reconstruction for pelvic Ewing’s sarcoma: a case report

**DOI:** 10.1186/s13256-024-04670-5

**Published:** 2024-07-29

**Authors:** Kazunori Nakayama, Seiji Shimomura, Toshiharu Shirai, Ryu Terauchi, Naoki Mizoshiri, Yuki Mori, Tomoki Saito, Yusei Katsuyama, Shinji Tsuchida, Kenji Takahashi

**Affiliations:** Department of Orthopaedics, Graduate School of Medical Science, Kyoto Prefectural University of Medicine, 465 Kajii-Cho, Kawaramachi-Hirokoji, Kamigyo-Ku, Kyoto, 602-8566 Japan

**Keywords:** Ewing’s sarcoma, Pelvic, Infection, Pelvic ring reconstruction

## Abstract

**Background:**

Ewing’s sarcoma is a primary bone tumor predominantly observed in children and adolescents, necessitating a multidisciplinary treatment approach. While localized cases have a 5-year survival rate of 60–70%, the prognosis is significantly worse in pelvic advanced cases with metastasis. Moreover, pelvic Ewing’s sarcoma has the unique problem of leading to high rates of postoperative infection.

**Case presentation:**

We present the case of a Japanese 14-year-old boy with left iliac Ewing’s sarcoma and multiple metastases. At the initial visit, imaging revealed a large tumor in the left iliac bone with extraosseous extension and metastasis to multiple sites. Neoadjuvant chemotherapy resulted in significant tumor reduction. Surgical resection was performed without pelvic ring reconstruction to enable early postoperative chemotherapy and minimize postoperative infection risk. Despite complete abductor muscle removal, the patient achieved a stable gait postoperatively by centering the load axis.

**Conclusion:**

Our case highlights the successful management of a left iliac Ewing’s sarcoma with multiple metastases, with a focus on functional preservation and infection risk reduction. Pelvic ring reconstruction was not performed to avoid postoperative complications, emphasizing the importance of early postoperative chemotherapy. The patient achieved a stable gait postoperatively, demonstrating the potential benefits of this approach in similar cases.

## Background

Ewing’s sarcoma is a primary malignant bone tumor in children and adolescents that requires multidisciplinary treatment, including chemotherapy, surgery, and radiation therapy [1, 2]. The 5-year survival rate of Ewing’s sarcoma is 60–70% in patients with localized disease and successful treatment, whereas in advanced cases with metastatic disease, the prognosis is poor, with a 5-year survival rate at the time of initial diagnosis of less than 30% [3]. The prognosis of patients with pelvic involvement is worse than that when extremities are affected [4]. Furthermore, postoperative infection rates of 35.6% [5] and 20.4% [6] have been reported in pelvic cases. Postoperative infection not only delays chemotherapy initiation but also affects prognosis. Therefore, during surgery for pelvic Ewing’s sarcoma, it is important to consider the balance between the postoperative function of the affected limb after resection and the reduction of postoperative infection risk. We report the case of a patient with left iliac Ewing’s sarcoma and multiple metastases who underwent multidisciplinary treatment and achieved good walking ability without postoperative reconstruction of the pelvic ring.

## Case presentation

**Fig. 1:**
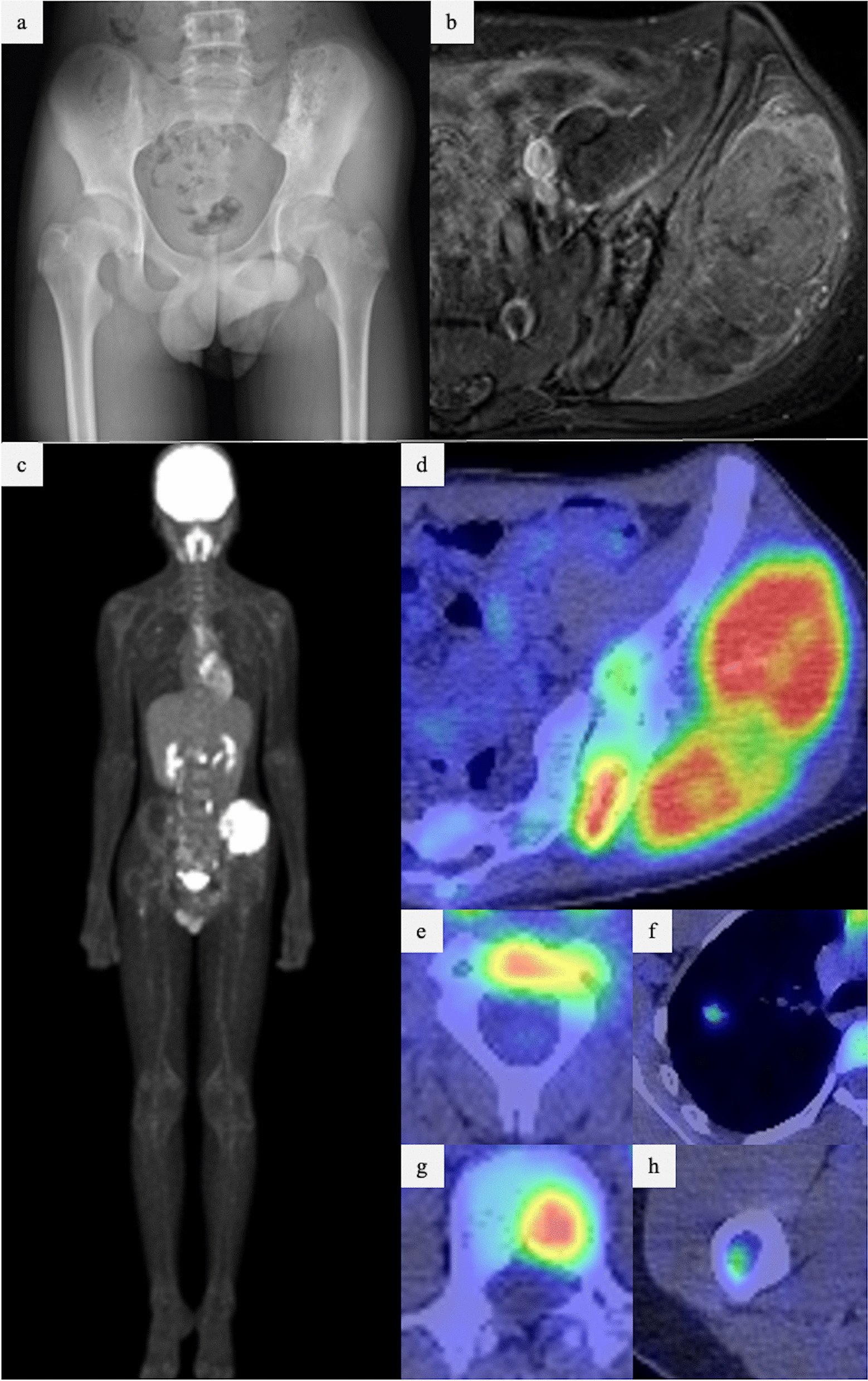
Imaging findings at the first visit to our hospital. **a** X-ray showing a tumor in the left iliac bone with osteosclerosis and osteolysis. **b** MRI showing a tumor in the left iliac bone, with a conspicuous extra-bone mass in the gluteus medius muscle. **c**–**h** PET–CT showing accumulation in the C2 vertebra (**e**), right lung (**f**), L2 vertebra (**g**), and right femur (**h**), in addition to the primary tumor (**d**). CT, computed tomography; MRI, magnetic resonance imaging; PET–CT, positron emission tomography–computed tomography

A 14-year-old Japanese boy was referred to our hospital with a suspected pelvic tumor. At the initial visit, a hard, painful, and tender bony mass was palpable on the left buttock. Blood testing indicated a mildly elevated inflammatory response (alkaline phosphatase 223 U/L, lactate dehydrogenase 321 U/L, C-reactive protein 1.79 mg/dL, and white blood cell count 7800/μL). Plain radiographs showed mixed osteosclerosis and osteolysis in the lateral iliac bone of the left sacroiliac joint (Fig. 1a). Magnetic resonance imaging (MRI) showed T1-weighted isointensity and T2-weighted heterogeneously high signal, while gadolinium contrast MRI showed the enhancement of a tumor in the left iliac bone with a large extraskeletal mass. The tumor diameter was 104 × 49 × 93 mm (Fig. 1b). Fluorodeoxyglucose positron emission tomography–computed tomography (PET–CT) images showed accumulations in the same area, the C2 and L2 vertebrae, right femur, and right lung (Fig. 1c–h). Computed tomography-guided biopsy of the iliac bone tumor was performed. Histological examination revealed small nuclei with a high nuclear-to-cytoplasmic ratio and monotonous atypical cells with increased chromatin in the perivascular area. The *EWS*–*FLI1* fusion gene was detected by fluorescence in situ hybridization. Therefore, the patient was diagnosed with primary Ewing’s sarcoma of the iliac bone.

**Fig. 2:**
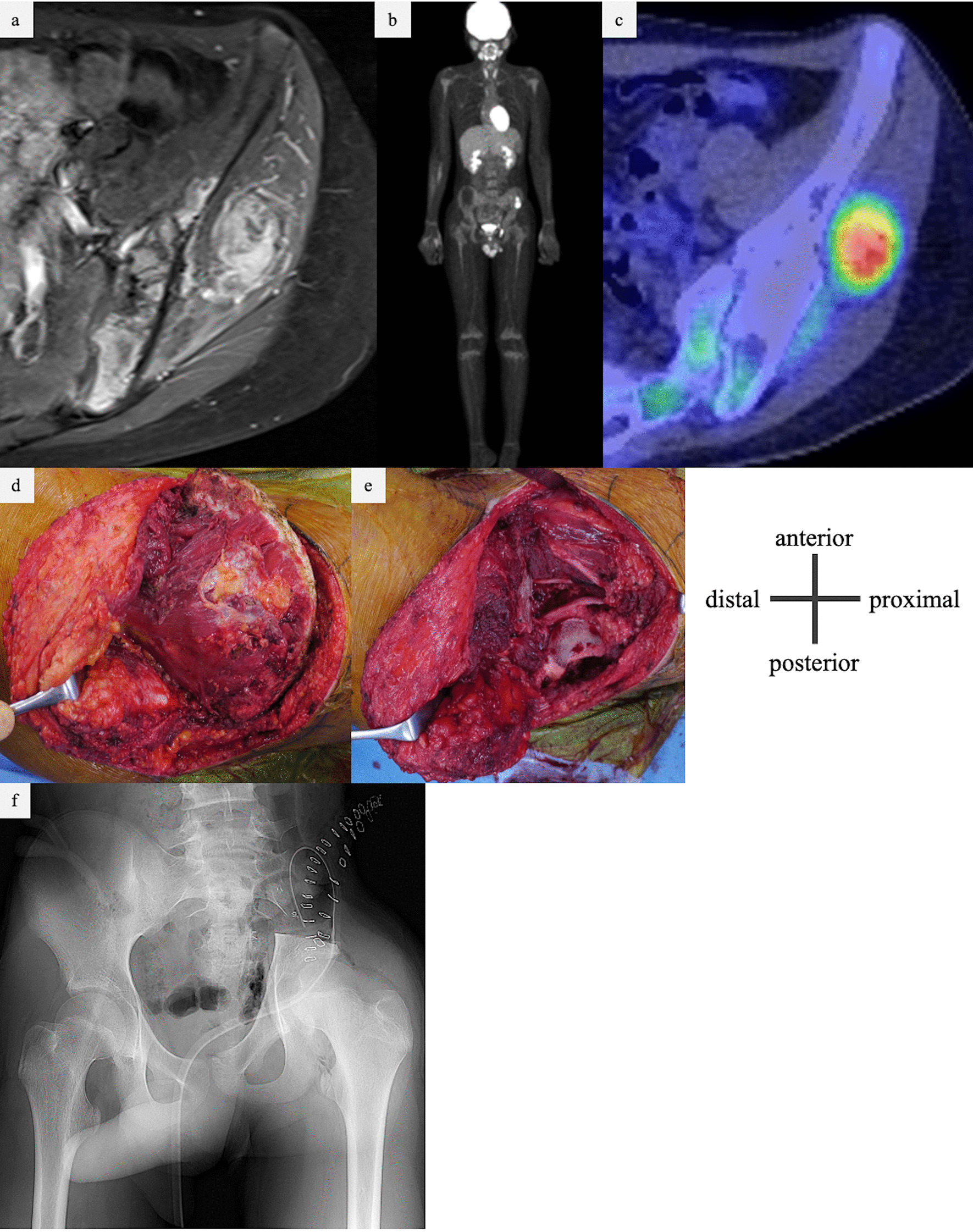
**a**–**c** Imaging findings after five courses of VDC/IE chemotherapy. **a** MRI showing the presence of an extra-bone mass within the gluteus medius muscle, although reduced in size. **b**, **c** PET–CT showing residual accumulation of primary tumor but disappearing of metastases. **d**, **e** Intraoperative findings of wide excision for the left iliac malignant bone tumor. **d** Before excision, an extra-bone mass was palpable within the gluteus medius muscle and resected together with the iliac wing. **e** Osteotomy was performed on the sacrum slightly medial to the sacroiliac joint and just above the acetabulum. **f** Postoperative X-ray. Pelvic ring reconstruction was not performed. PET–CT,  positron emission tomography–computed tomography; VDC/IE, Vincristine doxorubicin cyclophosphamide/Ifosfamide etoposide

The patient was treated with neoadjuvant chemotherapy with vincristine (2 mg/m^2^/day, doxorubicin 37.5 mg/m^2^ for 2 days, cyclophosphamide 1.2 mg/m^2^/day, ifosfamide 1.8 g/m^2^/day for 5 days, and etoposide 100 mg/m^2^/day for 5 days). MRI taken after preoperative chemotherapy showed that the extraosseous mass was reduced, with a tumor reduction rate of 90.5%. PET–CT scan after chemotherapy showed that there was almost no accumulation of left iliac tumor and metastases disappeared (Fig. 2a–c). According to the Response Evaluation Criteria in Solid Tumors guidelines version 1.1, the target lesion showed a partial response [104 × 49 mm → 20 × 24 mm (90.5% reduction)], and the overall judgment was of complete response (the multiple metastases disappeared). Therefore, the overall effect was determined to be of partial response.

**Fig. 3:**
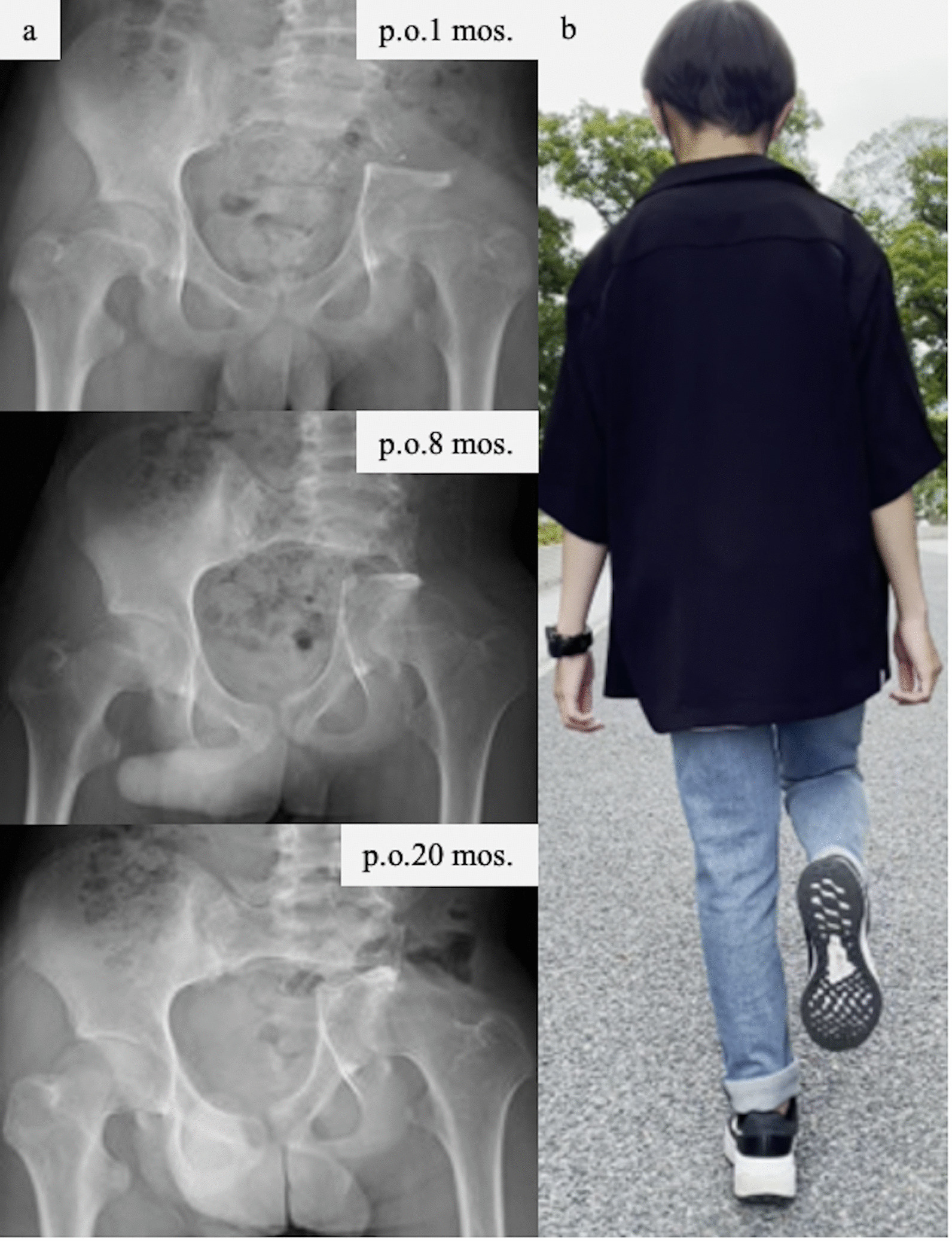
**a** Progress of postoperative plain X-ray imaging. The patient’s left hip was gradually centralized to the pelvic center. **b** There was no remarkable Trendelenburg sign at 20 months after the excision

The day before surgery, the nutrient artery and superior gluteal artery were embolized using transcatheter arterial embolization. Wide excision of the iliac Ewing’s sarcoma was performed. During surgery, osteotomy was performed on the sacral side of the sacroiliac joint and the cephalic side of the acetabulum, and combined excision of the iliac, gluteus minimus, and gluteus medius muscles was performed. Thereafter, pelvic reconstruction was not performed (Fig. 2d–f). The operative time was 2 hours 36 minutes, and the blood loss was 336 mL. Postoperative rehabilitation consisted of unloading until 3 weeks postoperatively, followed by partial loading and full loading at 6 months postoperatively. The postoperative course was uneventful, with no apparent complications such as infection. Postoperative chemotherapy was resumed on the 20th day after surgery. His left hip shifted medially and posteriorly owing to the flexibility of the pubic symphysis, and gradual centralization of the hip occurred postoperatively. Eight months after surgery, the hip joint shifted just below the spinal axis (Fig. 3a). At 19 months postoperatively, the patient was painless, Trendelenburg’s sign was mild, and the patient was able to stand stably on one leg and walk smoothly (Fig. 3b).

## Discussion and conclusions

In this case, we did not choose pelvic ring reconstruction, which is associated with frequent postoperative adverse events such as infection in patients with pelvic Ewing’s sarcoma with poor prognosis, in order to resume chemotherapy early. Although the patient required a supplemental height implant, Trendelenburg’s sign was mildly suppressed, and the patient was able to walk independently and stably from a functional viewpoint.

Poor prognostic factors for Ewing’s sarcoma include pelvic origin, metastasis at initial diagnosis, failure to respond to chemotherapy, and a tumor volume of 200 mL or more [7]. The poor prognostic factors in this case were pelvic origin, metastasis at the time of initial diagnosis, and a tumor volume of > 200 mL. Therefore, this case was classified as having a poor prognosis. Chemotherapy has been shown to be effective in Ewing’s sarcoma. It has been shown that intensive chemotherapy administered every 2 weeks has a better prognosis than standard chemotherapy administered every 3 weeks [8]. Therefore, we consider that early chemotherapy is important even after surgery in cases with a poor prognosis, as in this case.

During surgery for malignant pelvic tumors, whether to perform pelvic ring reconstruction after wide excision is controversial. The advantages of pelvic ring reconstruction include stabilization of the pelvic ring and prevention of leg length inequality and scoliosis. However, there is a risk of infection due to the extended operative time associated with reconstruction to insert implants and bone grafts. Severyns *et al.* reported an infection rate of 35.6% (16/45 cases) after surgery for pelvic malignancies [5]. Additionally, Angelini *et al.* reported a significantly lower postoperative infection rate in patients with pelvic malignancies who did not undergo pelvic ring reconstruction (15%; 20/133 patients) than in those who did (26%; 35/137 patients) [6]. Moreover, postoperative infection causes delays in postoperative chemotherapy. Delayed resumption of postoperative chemotherapy has been reported to affect prognosis. According to Imran *et al.*, patients who started chemotherapy on or after the 21st postoperative day had a worse prognosis than those who started chemotherapy earlier [9]. In patients with poor prognosis, such as this case, early postoperative chemotherapy is essential. Therefore, reconstructive surgery was not performed to reduce the risk of postoperative infection.

In wide excisions of malignant pelvic bone tumors, such as in this case, the gluteus medius is often resected. When the gluteus medius is resected, hip abduction is impaired, resulting in the Trendelenburg sign and claudication. Beadel *et al.* reported that patients who did not undergo pelvic ring reconstruction had shorter operative time, less blood loss, fewer operative complications, equal local and systemic tumor control, and equal or better functional outcomes than patients who underwent pelvic reconstruction [10].

**Fig. 4:**
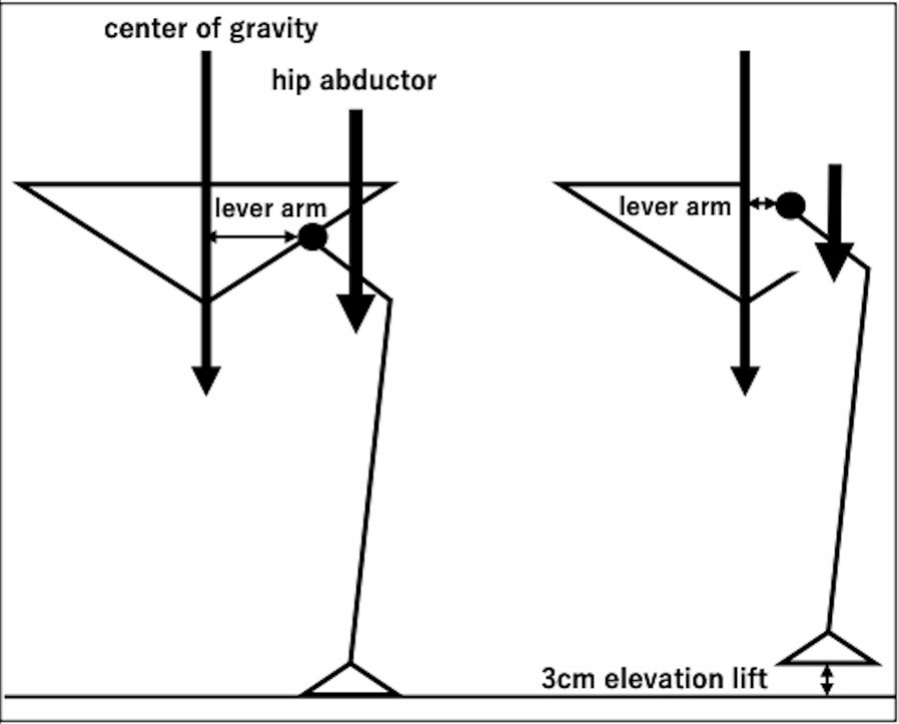
Schematic representation of this case. Centralization of the left hip joint without pelvic ring reconstruction shortened the lever arm between the spine and hip joint. Therefore, lower abductor muscle strength is required for stable walking. This figure is our own work

The unique technique in this case was to preserve the acetabulum unlike the common hip transposition. This surgery preserved the hip joint, which resulted in a change in the alignment of the pelvis. In this case, in addition to the P1 segment resection, the gluteus medius and gluteus minimus were also resected, which was expected to result in a significant loss of abductor muscle strength. After resection of the pelvic tumor, intentional nonreconstruction of the pelvic ring but preserving the acetabulum resulted in the centralization of the hip joint. The lever arm between the center of load and the hip joint during standing was shortened, allowing the patient to walk stably and stand on one leg with a less evident Trendelenburg sign, even though the abductor muscles, gluteus medius, and gluteus minimus were completely removed (Fig. 4). If the acetabulum is excised, the hip joint cannot be centralized, leading to a less stable gait due to the dislocation of the femoral head within the gluteal muscles.

Since the patient is young, the flexibility of soft tissues such as the pubic symphysis and the fact that the acetabular capsule will become larger with future growth may also be advantageous. In such cases, where the abductor muscle must be removed in addition to the P1 segment for a malignant pelvic tumor, it may be useful to preserve acetabulum and avoid pelvic ring reconstruction, for centralization of the axis of loading to gain function of the affected limb.

Herein, we report a case of left iliac Ewing’s sarcoma with multiple metastases that resulted in good walking ability without pelvic ring reconstruction. Early postoperative chemotherapy was crucial, and pelvic reconstruction was not performed to reduce the risk of infection. This allowed centralization of the hip joint on the patient’s side and a stable gait without abductor muscles. This technique may be useful for the P1 resection of malignant pelvic tumors.

## Data Availability

The datasets used and analyzed in the current study are available from the corresponding author upon reasonable request.

## Abbreviation

MRI: Magnetic resonance imaging
PET–CT: Positron emission tomography–computed tomography
CT: Computed tomography
